# Mechanisms of Transmission and Processing of Pain: A Narrative Review

**DOI:** 10.3390/ijerph20043064

**Published:** 2023-02-09

**Authors:** Girolamo Di Maio, Ines Villano, Ciro Rosario Ilardi, Antonietta Messina, Vincenzo Monda, Ashlei Clara Iodice, Chiara Porro, Maria Antonietta Panaro, Sergio Chieffi, Giovanni Messina, Marcellino Monda, Marco La Marra

**Affiliations:** 1 Department of Experimental Medicine, University of Campania “Luigi Vanvitelli”, 80138 Naples, Italy; 2 Department of Psychology, University of Campania “Luigi Vanvitelli”, 81100 Caserta, Italy; 3 Department of Movement Sciences and Wellbeing, University of Naples “Parthenope”, 80133 Naples, Italy; 4 Department of Clinical and Experimental Medicine, University of Foggia, Viale Pinto, 71100 Foggia, Italy; 5 Department of Biosciences, Biotechnologies and Biopharmaceutics, University of Bari, 70125 Bari, Italy

**Keywords:** nociceptive information, pain, neurobiology, neurons, inflammatory diseases

## Abstract

Knowledge about the mechanisms of transmission and the processing of nociceptive information, both in healthy and pathological states, has greatly expanded in recent years. This rapid progress is due to a multidisciplinary approach involving the simultaneous use of different branches of study, such as systems neurobiology, behavioral analysis, genetics, and cell and molecular techniques. This narrative review aims to clarify the mechanisms of transmission and the processing of pain while also taking into account the characteristics and properties of nociceptors and how the immune system influences pain perception. Moreover, several important aspects of this crucial theme of human life will be discussed. Nociceptor neurons and the immune system play a key role in pain and inflammation. The interactions between the immune system and nociceptors occur within peripheral sites of injury and the central nervous system. The modulation of nociceptor activity or chemical mediators may provide promising novel approaches to the treatment of pain and chronic inflammatory disease. The sensory nervous system is fundamental in the modulation of the host’s protective response, and understanding its interactions is pivotal in the process of revealing new strategies for the treatment of pain.

## 1. Introduction

Knowledge about the transmission and processing mechanisms of nociceptive information, both in healthy and pathological states, has greatly expanded in recent years. This rapid progress is due to a multidisciplinary approach, involving the simultaneous use of different branches of study, such as systems neurobiology, behavioral analysis, genetics, and cell and molecular techniques. Pain is necessary for the survival and maintenance of the integrity of organisms. In fact, pain-induced behavioral changes lead an organism to avoid harmful stimuli in future encounters. It is clear that the interactions between the nervous and immune systems are closely linked through molecular and cellular interactions in the process of pain sensation. However, prolonged or chronic pain can result in secondary symptoms, such as anxiety and depression, and cause a decrease in the overall quality of life. The transmission of pain is linked to nociceptors, which are a specialized subset of sensory neurons that mediate pain and densely innervate peripheral tissues. Various subsets of nociceptors are further divided according to the type of stimuli (mechanical, chemical, thermal, or noxious) they respond to [1]. Nociceptors are predominantly made up of nerve terminals that express both ligand and voltage-gated ion channels [2]. Nociceptor neuron activity and pain sensitivity can be modulated by immune cells that release mediators. Immune cells, in turn, can be modulated by the nociceptors that release neuropeptides and neurotransmitters that act on innate and adaptive immune cells. In this way, the immune response is influenced by neural signaling, and consequently, this neural signaling contributes to the development of local and systemic inflammatory diseases.

## 2. Characteristics and Properties of Nociceptors

### 2.1. Structural Characteristics

When interacting with the external environment, living organisms must be able to clearly recognize harmful stimuli and react to them in an appropriate way. This important task is carried out by the nociceptors that make up a part of the somatosensory nervous system. These nociceptors respond to harmful or potentially tissue-damaging stimuli and transmit stimuli from the skin, muscles, joints, and viscera [3]. Nociceptors are classified according to the characteristics of their axons, which are generally divided into two categories: unmyelinated (C fibers) or slightly myelinated (Aδ fibers). The soma of nociceptors are commonly small in diameter in both the dorsal pathway and in other sensory ganglia. Neurons with small soma diameter usually have myelinated Aδ fibers or unmyelinated C fibers, while Aα/β-fibers can be found on cells of larger dimensions. Soma diameter and axon myelination are not associated with nociceptor functionality [4]. In fact, the soma of non-visceral nociceptors are smaller than those of visceral nociceptors, but this difference in size has no bearing on their respective degrees of function [4].

### 2.2. Stimuli

Nociceptor activation is determined by the pain stimulus: this depends on the site of generation and mode of activation. The site of application of the stimulus is important because it can influence the intensity of the nociceptor response. An interesting example is that of corneal nociceptors, which are activated by weaker stimuli than skin nociceptors are [5]. The nature of the stimulus is also important. Stimuli brought about by cutting or crushing, for example, activate most skin nociceptors but do not activate those in the joints, muscles, or viscera, which instead quickly respond to other types of mechanical forces, such as rotation and distention [5]. In addition to cutting and crushing injuries, harmful stimuli that are able to activate nociceptors in the skin also include chemical, thermal, and mechanical damage. The most nociceptor-rich tissue is the skin, which contains several different types of nociceptors [6].

### 2.3. Nociceptor Population

The most expressed nociceptor in the skin is the polymodal nociceptor. It is capable of responding to several kinds of stimuli, but it is not the only type of nociceptor found there. In fact, the skin contains a wide variety of nociceptors that differ in selective modality (such as C-heat nociceptors and C-mechano-cold nociceptors) [7]. In other tissues, however, the two main types of harmful stimuli that are detected are chemical (changes in pH or inflammation) and mechanical (torque or distention) changes. Several studies on the nociceptors found in joints use mechanical stimuli and demonstrate that there are sub-populations of mechanical nociceptors that are sensitive to both low- and high-intensity stimuli. Many of these nociceptors, both low-threshold and generally all high-threshold joint and visceral mechanoreceptors, are polymodal. They transduce the harmful stimuli and then sensitize the organism [8], which leads to the understanding that low-threshold afferents contribute to the onset of pain during tissue inflammation. Most nociceptors are activated by mechanical stimuli, but various subpopulations with different sensitivities to other and additional noxious stimuli have been described in other species. Mechanonociceptors are only activated by mechanical stimuli. If they also respond to other stimuli, such as thermal stimuli, they are then called polymodal nociceptors but can be further subclassified according to their specific sensitivity to noxious thermal stimuli, for example, to heat and/or cold [9,10]. Various nociceptor subpopulations and acute pain sensitivity have been thoroughly studied and are now understood. However, the most studied receptor involved in nociception is the large family of transient receptor potential (TRP) channels, which is comprised of six subfamilies: TRP vanilloid (TRPV), ankyrin (TRPA), mucolipin (TRPML), polycystin (TRPP), canonical (TRPC), and melastatin (TRPM) [11].

The TRPV1 receptors are expressed on C-fiber nociceptors and detect pain stimuli associated with noxious heat and the development of heat hyperalgesia [12,13]. TRPV1 receptors are also involved in the detection of noxious pinch stimuli. They are activated by capsaicin, camphor, allicin, low pH, and hypertonicity [11,13]. Many factors, including physiological processes and natural ligands, such as reactive oxygen species (ROS), low pH, allyl isothiocyanate, tetrahydrocannabinol, allicin, gingerol, and methyl salicylate can activate TRPA1 receptors [12]. TRPA1 receptors are activated by calcium [14] and are activated and sensitized by inflammatory factors (such as bradykinin) in dorsal root ganglion neurons [12]. The transient receptor potential cation channel subfamily melastatin member 8 (TRPM8) is also associated with the detection of noxious cold stimuli and contains a C-fiber that expresses both cold- and menthol-sensitive ion channels [15]. Moreover, C-fiber nociceptors that express the Mas-related G-protein-coupled receptor D transduce noxious mechanical stimuli applied with a blunt probe, such as von Frey hair [16], whereas A-fiber mechanonociceptors detect sharp and potentially tissue-damaging mechanical stimuli, such as a pinprick. These fibers are characterized by the expression of the neuropeptide Y receptor type 2 [17].

Another receptor involved in nociception is the anoctamin 1 (ANO1) receptor, a calcium-activated chloride channel. It can be activated by noxious heat stimuli, which, in turn, induce a burning pain sensation through the release of inflammatory factors such as bradykinin from dorsal root ganglia neurons [11]. Communication and cohesive action between ANO1 receptors and TRP channels in the generation of strong pain and the regulation of neuronal excitability has been suggested [18].

Piezo channels are mechanosensitive receptors that are made up of two cationic channels, Piezo1 and Piezo2, that are relatively homologous in structure [19]. Piezo channels are expressed in various tissues and can be activated by several types of mechanical stimuli, such as stretching, pushing, pulling and exposure to hypo-/hyper-osmotic solutions [20]. Piezo2 channels show faster kinetic properties than Piezo1 channels and mediate a rapid membrane response. These channels seem to be more specific for the detection of transient mechanical forces. Piezo1 channels have slower kinetic characteristics and react to more persistent activation. Both types of channels, however, mediate somatic and visceral pain [21].

### 2.4. Sensitization

One of the characteristic properties of nociceptors is their ability to cause sensitization, which is the capability to increase neuronal excitability. Sensitization is a process that consists of a reduction in the threshold of activation, as well as an increase in the response rate to harmful stimulation. It usually results from tissue insult and inflammation [22]. Moreover, stimuli that do not generate an effect before the process of sensitization takes place may subsequently become effective and develop spontaneous activity after sensitization occurs [23]. The adaptive response can be reduced by nociceptor sensitization, which can be observed when the stimulus application is prolonged [24]. Sensitization is a central property for nociceptor neurons, but it is not a process that is specific to them. Sensitization may be associated with afferents that encode other sensory modalities as well [25].

### 2.5. Efferent Function

Another property of nociceptor neurons is their efferent function. It is important to note that only some nociceptors, for example, peptidergic nociceptors, have this function and are capable of releasing substances from their peripheral terminals. This characteristic serves to guarantee the maintenance of tissue integrity in the absence of tissue damage. For instance, nociceptive nerves are required for enforced hematopoietic stem cell (HSC) mobilization, and they collaborate with sympathetic nerves to maintain HSCs in bone [26]. Neurogenic inflammation may be generated from an increase in the peripheral release of afferent transmitters during sterile inflammation such as that associated with migraines [27]. Therefore the release of molecules from nociceptors is not exclusively associated with the process of inflammation but collaborates in order to lead to the pain associated with tissue damage [28]. The role of the back-propagation of afferent activity across collateral branches was initially described as singularly mediating the peripheral release of afferent molecules, but some studies demonstrate that there may also be a secondary contribution from the antidromic activity of the spinal cord in this scheme [29,30]. Neurogenic inflammation and pain caused by tissue damage may be alleviated by the inhibition of voltage-gated Ca^2+^ and Na^+^ channels which are involved in the peripheral release of afferent transmitters [31,32]. Nociceptors cannot be identified by a single criterion, which is why they do not belong to a homogeneous group of afferents. There are many anatomical, biochemical, physiological, and functional variations between them. Visceral pain and other discomfort, such as hypersensitivity to organ filling, acidic or burning pain, and the sensation of bloating, may be due to subpopulations of visceral nociceptors [4,33]. The possibility of introducing new therapeutic agents must be inclusive and, therefore, overcome the heterogeneity of the nociceptor subpopulations. This heterogeneity may be one of the reasons why introducing new therapeutic agents in the treatment of pain has proven to be so difficult and accounts for the many failures encountered in the use of new drugs and therapies in this line of treatment.

## 3. Clinical and Preclinical Studies on Ion Channel Signaling Modulation

Our understanding of the mechanisms regulating nociceptive processing has not yet produced an effective alternative to opioids [34] in the treatment of chronic pain. The abuse of these drugs, however, is a growing phenomenon [35]. The goal of pain treatment is to develop effective drug therapies with acceptable side effect profiles and minimal risk of abuse. To date, advances in pain biology have produced remarkable insights, and clinical and preclinical studies are now focusing primarily on the modulation of ion channel signaling [36]. Ion channels are the targets of most currently available pain medications and were discovered more than a decade ago [37,38]. Among these medications is carbamazepine, which acts by blocking sodium, calcium, and GABA channels and produces pain relief by blocking synaptic transmission. Similarly, lidocaine [39] is commonly employed as a local anesthetic and is used in surgical procedures to eliminate acute pain or treat chronic pain [39]. These anesthetics are administered at relatively high doses to block voltage-gated sodium channels, as well as potassium and calcium channels [40]. Gabapentin, originally developed for the treatment of epilepsy [41], is also currently used in the treatment of neuropathic pain. As a lipophilic analog of GABA, it interacts with the α2δ subunit of voltage-gated calcium channels [42]. Subsequent pharmacological research has resulted in the development of pregabalin [38], which has become the gold standard for the treatment of chronic pain associated with diabetic neuropathy.

The safety profile of nonselective agents, however, limit their continued use [43]. Nonselective ion channel blockers have functional consequences, especially if they result in the additional inhibition of ion channels other than those expressed in nociceptors, such as those expressed in the heart and central nervous system, for example. A more recent understanding of the specific sodium channels that are expressed on nociceptors has prompted the scientific community to search for selective inhibitors. This process has generated high-quality data on Nav isoforms [43]. It has been shown that congenital insensitivity to pain (CIP) can be conferred by mutations in Nav1.7 [37]. Subsequently, compounds capable of acting on these ion channels have entered clinical trials for pain. These compounds include proline derivative 5, a spiro-oxindole compound [44], and a series of sulfonamide aminoheterocycles [36]. To date, efficacy in treating pain in patients with congenital erythromelalgia has been reported, whereas no efficacy was found in a study of the treatment of pain in post-herpetic neuralgia. In addition, positive data were found in a study of trigeminal neuralgia and the reduction of pain from lumbosacral radiculopathy [36]. In addition to Nav 1.7, great interest has also been aroused by Nav1.8 as a potential target for the management of inflammatory and neuropathic pain [45,46].

TRP channels are cation channels involved in pain perception and thermosensation [47]. TRPV1 is activated by numerous stimuli, including heat (>42 °C), vanilloids, lipids, and protons/cations. Several highly selective TRPV1 antagonists are currently in clinical development for the treatment of pain. Although the use of desensitizing TRPV1 agonists reduces pain sensitivity [48,49], recent clinical trials have shown that blocking TRPV1 also affects body temperature. This unfortunate side effect has halted much of the drug development activity targeting this channel. Topical application, however, has been shown to be effective in preventing the initial pain flare-up that occurs with agonist-induced nociceptor excitation prior to desensitization. TRPM8 is activated in vitro by cold temperatures (10–23 °C) and cooling agents such as icilin and menthol. Researchers have recently revealed that the TRPM8 antagonist 15 produces an analgesic effect in experimental models of cold pain in humans without affecting core body temperature [50]. TRPA1 is activated by several ligands, such as cinnamaldehyde, endogenous ligand 4-hydroxynonenal (4-HNE) and the allyl isothiocyanate [41]. It is activated by noxious cold (<17 °C), [47] and has been directly linked to pain by a gain-of-function mutation causing familial episodic pain syndrome. The TRPA1 antagonist has been shown to be effective for the treatment of diabetic peripheral neuropathy and inflammatory conditions. TRPV3 is activated by camphor, 2-Aminoethoxydiphenyl borate (2-APB), and warm temperatures (>32 °C). To date, several patent applications have been filed for TRPV3, including pyridopyrimidine 2131 and benzimidazole [48]. In recent years, several selective TRPV4 antagonists have been shown to be effective, especially in the treatment of pulmonary edema. TRPV4 is activated by anandamide, 5,6-epoxyeicosatrienoic acid, GSK1016790A, and heat (27–34 °C). Increasing interest in the treatment of neuropathic pain has been placed on the Cav2.2 N-type subtype [51,52]. Ziconotide, an analgesic peptide derived from Conus magus snail toxin, requires intrathecal administration in order to be effective and is associated with significant side effects [53]. However, progress has been made in the development of N-type inhibitors.

T-type calcium channel blockade has also been shown to be effective in reducing neuropathic, inflammatory, and visceral pain. Phase 1 and 1b studies have demonstrated safety and good tolerability. Kv7 ion channels have also been widely investigated, given the strong genetic association between mutations in Kv7.2 and Kv7.3 channels and neuronal hyperexcitability [54]. Several preclinical models of pain [55,56] support the role of Kv7 channels in pain signaling. Retigabine was approved for use in partial-onset seizures and subjected to a Phase 2 proof-of-concept clinical trial for post-herpetic neuralgia pain (however, it was not shown to be superior to the placebo). Lupirtine (which is structurally similar to retigabine) has been approved in Europe for the treatment of lower back pain. Ionotropic glutamate receptor (iGluR) antagonists, particularly the subtype-selective GluN2Bs, have also attracted the attention of the pharmaceutical research community for the treatment of central nervous system disorders, including stroke and Parkinson’s disease [57]. These include ifenprodil [58] and the more selective derivative traxoprodil [59]. While the modulation of ion channel signaling by small molecules has demonstrated the ability to treat pain, current research is also considering the role of drugs acting on GABA, alpha2 adrenergic receptors, opioid receptors, and angiotensin 2 and Toll receptor antagonists, as well as adenosine and purine receptor agonists/antagonists. Additional targets being considered for study include lipid mediators and anti-inflammatory cytokines.

## 4. Immune System and Pain

**Figure 1:**
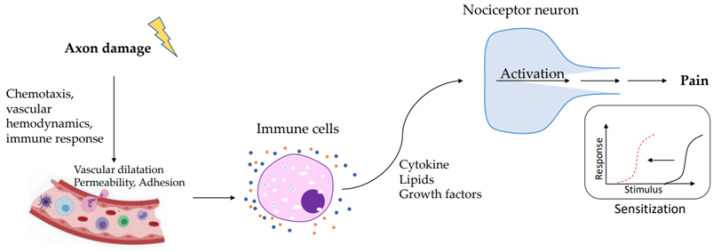
During axon damage, neuronal factors released from nociceptor sensory neurons directly drive leukocyte chemotaxis, vascular hemodynamics, and the immune response. Immune cells release mediators that are detected by receptors of the nociceptor peripheral nerve that transduce the stimuli to produce pain sensitization.

The pain channeled by nociceptive neurons is influenced by the immune system through the release of molecular mediators which sensitize the nociceptors and are intimately coupled to cause increases in the sensation of pain. During inflammation, molecular mediators are released and are subsequently detected by nociceptors on peripheral nerve terminals. Moreover, numerous soluble mediators may also be secreted and can amplify the recruitment of immune cells. Edema and hyperemia, for example, are caused by molecules such as calcitonin gene-related peptide, substance P, bradykinin, and nitric oxide, which are released during axon damage [60]. The vascular changes caused by the action of these molecules allow for invasion by circulating immune cells that further release molecular mediators. After activation, action potentials are transduced to nociceptor cell bodies in DRG. The stimuli are then relayed to the spinal cord and brain which process the stimuli as pain. During inflammation, action potentials require lower activation stimuli because the threshold for nociceptor neurons to fire becomes reduced in this process. This lower required threshold leads to pain sensitivity or ‘hyperalgesia’ (Figure 1). Central glia respond to peripheral injury [61,62,63,64] through afferent nerve input [65,66,67], circulating cytokines [68], and interaction with immune cells [69,70]. Consistent activation of glia has been observed in studies of deep tissue injury from different districts, such as muscles [65,71], joints [72], nerve trunks [73], and viscera [74,75], with hyperactivity being directly related to inflammatory injury and pain intensity. In addition, glia selectively promote sensitization following injury. Not all forms of nociceptive input regulate glial function, however. Glial responses are selective for different forms of primary afferent input. Among the prototypical proinflammatory cytokines, interleukin-6 (IL-6) has been shown to act as a messenger for the transmission of peripheral immune signals to the central nervous system by inducing COX-2 activity and PGE2 release from cerebral vascular endothelial cells [76,77]. The migration of immune cells into the central nervous system is selective. In fact, peripheral macrophages or monocytes spread into the spinal cord and differentiate into cells with a microglial phenotype [78]. These cells then directly contribute to glial responses in the central nervous system. Upon the arrival of a nociceptive nerve impulse, neural and immune mediators, such as glutamate, ATP, substance P, calcitonin gene-related peptide, brain-derived neurotrophic factor (BDNF), IL-6, and C-C motif chemokine ligand 2 (CCL2), are released. These act on receptors present on the postsynaptic nerve terminal and on microglia and astrocytes, which modulate glial activity [79,80,81,82,83,84,85]. Neurons regulate microglia activity through multiple cellular pathways. A recent study found that a microglia-specific signaling pathway is mediated by neuregulin-1 (NRG-1) [86], which leads to the activation of spinal microglia, the release of proinflammatory cytokines (including interleukin-1β), chemotaxis, and the subsequent development of pain hypersensitivity [86]. Furthermore, microglia have been shown to be somewhat involved in pain suppression. Astrocyte activation is also modulated by neuronal activity following peripheral injury [65].

Both microglia and astrocytes release substances that can influence neuronal activity. Activated microglia release various mediators that act on neurons and sensitive nociceptors [82,87,88,89]. Astrocytes, on the other hand, are uniquely positioned to interact with neurons in order to regulate their synaptic activity. Release of glutamate at nerve terminals activates metabotropic glutamate receptors on astrocytes, increasing Ca^2+^ mobilization. This leads to the release of a variety of mediators, including glutamate, D-serine, and ATP, which, in turn, modulate neuronal activity [90]. N-methyl-D-aspartate receptor (NMDA) receptors play an important role in synaptic plasticity and persistent pain. D-serine, an NMDA receptor co-agonist, acts on synaptic NMDA receptors, while astrocytic glutamate binds to extrasynaptic NMDA receptors. The astrocytic glutamate transporter (GLT-1) buffers the glutamate that is released at synapses in order to prevent excessive activation of postsynaptic glutamate receptors. However, GLT-1 is downregulated after injury [91], and glutamine transport between astrocytes and neurons is altered [92,93]. These changes in synaptic glutamate homeostasis lead to increased dorsal horn excitability and contribute to the development of persistent pain [94,95]. Among the numerous immune-derived or glia-derived mediators that are involved in pain hypersensitivity, interleukin-1β (IL-1β) is a key cytokine that modulates microglia, astrocytes, and neurons [96,97]. Interleukin-1 receptor activation facilitates the phosphorylation of NMDA receptors, thereby inducing changes in synaptic strength and causing behavioral hyperalgesia [63,82,95]. Following injury, tumor necrosis factor alpha (TNF-α) is also upregulated in pain pathways and is secreted by both immune and glial cells [61,63]. In the rostral ventromedial medulla, which is responsible for the descending modulation of pain, TNF-α facilitates the phosphorylation of NMDA receptors and stimulates the phosphorylation of the GluA1 subunit of the AMPA receptor as well as its trafficking to the membrane in dorsal horn neurons [98]. These results support the idea that glia-derived pro-inflammatory cytokines interact with excitatory amino acid receptors. Inflammatory conditions, such as inflammatory bowel disease and rheumatoid arthritis, are accompanied by chronic pain. The resolution of the tissue immune response in these conditions leads to the reduction of pain, which demonstrates the importance of the immune system in neuronal sensitization.

## 5. Immune Regulation of Pain

Nociceptive neurons express several receptors, including those for immune cell-derived cytokines, protease growth factor, and lipids. After linking with its ligand, these receptors activate a signaling cascade that modifies the permeability of ion channels, and these changes lead to an increase in neuronal firing [99]. The role of immune mediators in pain sensitivity has come to light in several recent studies. Neutrophils migrate to the tissue where they support the propagation of pain through the production of cytokines and prostaglandin E2 (PGE2). This process has been demonstrated in murine models of both neuropathic pain [100] and carrageenan-induced inflammatory pain [101]. Myeloid cells are responsible for pain sensitization in incisional injury [102]. Other immune cells, such as mast cells, play a role in sensitizing receptors. After activation, mast cells release granules and cytokines (interleukin-5, tumor necrosis factor alpha, IL-6, and IL-1β), serotonin, histamine, and nerve growth factor. These factors induce the sensitivity to pain on nociceptors [103,104,105]. Mast cells are involved in inflammation both acutely [106] and chronically. In fact, mast cells contribute to the chronicity of pain [107]. Macrophages act as sentinel myeloid cells. They are present everywhere in the body and are recruited to inflammatory sites during tissue injury. The role of macrophages and monocytes, which is to produce several cytokines, growth factors, and lipids that influence the nociceptive neurons in pain sensation in disease conditions characterized by chronic pain, has been expressed in several studies [108,109,110,111,112]. Nociceptors may also be sensitized by T cells that release IL-17A and IFN-γ, which act at nerve terminals to increase neuropathic pain [113]. It has been shown, in recent years, that mesenchymal stromal cells (MSCs), which have the ability to differentiate into distinct mesenchymal cell lineages, including chondrogenic, adipogenic, and osteogenic lineages, [114,115], are able to modulate the inflammatory response through interleukin-10 (IL-10). During inflammation, different regulatory immune cells are induced, and their population is expanded. IL-10, which induces these regulatory immune cells, plays a critical role in immunomodulation by MSCs. In this scenario, an understanding of the effects that MSCs have on regulatory immune cells, as well as a deep comprehension of their potential manipulation in order to induce tolerance, could serve as a novel therapeutic approach to pain management [116].

## 6. Cytokines Involved in Pain

Cytokines derived from immune cells during inflammatory states play a key role in nociceptor activity and pain sensitization. The role of cytokines has been well described in previous studies [117] (Table 1).

IL-1β is the first cytokine described [118] as having hyperalgesic activity [119]. This is a crucial point of neuroimmunology because it demonstrates that a molecule secreted by the immune system can induce neuronal sensitization and consequently proves that an antagonist of the receptor can inhibit the hyperalgesic effect [120]. The role of cytokines in pain modulation has been demonstrated in the treatment of several diseases, including arthritis, neuropathic pain, and cancer-related pain [121,122]. The role of IL-1β, IL-6, tumor necrosis factor alpha, IL-17A, and interleukin-5, which act directly on nociceptor neurons, has been demonstrated. The sensitization of nociceptors from IL-1β is carried out through the phosphorylation of p38 mitogen-activated protein kinases (MAPK) on Nav1.8 sodium channels. This translates to an increase in action potential generation and results in mechanical and thermal hyperalgesia [123]. The role of IL-1β in pain sensitivity to thermal stimuli is due to the activation of interleukin 1 receptor type 1 (IL-1R1) on nociceptor neurons, which, in turn, increases TRPV1 expression [124]. Inflammatory pain is increased by IL-6, which induces prostaglandin production [118] and increases TRPV1 and transient receptor potential ankyrin 1 (TRPA1) expression by binding to its receptor gp130, which is expressed on nociceptors [125,126]. The inflammatory pain induced by TNF/α in vivo is linked to both tumor necrosis factor receptor 1 and prostaglandins [127,128]. Moreover, nociceptor sensitivity is influenced by TNF/α because TNF/α is also active in the p38 MAPK phosphorylation of Nav1.8 and Nav1.9 sodium channels and, therefore, alters neuron excitability [129,130]. These studies affirm that IL-1β, IL-6, and TNF/α induce prostaglandin synthesis and/or improve the activation of TRP and Nav channels and that this activation leads to the sensitization of nociceptor neurons. The role interleukin-17A (IL-17A) plays in autoimmune disease is interesting as this cytokine is richly expressed in nociceptive neurons. The pain linked with autoimmune diseases, such as arthritis and psoriasis, is associated with IL-17A, which induces a fast increase in neuronal excitability, and implicates the involvement of this molecule in the evolution of these disease processes [124]. Moreover, IL-17A induces a hyperalgesia which is dependent on the amplification of TNF/α, IL-1β, chemokine (C-X-C motif) ligand 1 (CXCL1), endothelin-1, and prostaglandins in antigen-induced arthritis [131].

## 7. Lipids Involved in Pain

The most widely used drugs to inhibit the process of inflammatory pain are nonsteroidal anti-inflammatory drugs (NSAIDs). Their mechanism of action is based on the inhibition of cyclooxygenases (COX), which are involved in the production of prostanoids (prostaglandins, prostacyclins, and thromboxane). PGE2 plays a key role in inflammatory pain. The action of PGE2 is mediated by rhodopsin-type receptors, which are characteristic of PGs. There are various receptors for PGE2: EP1, EP2, EP3, and EP4. They are codified by different genes and are expressed differently in different tissues [132]. After activation, the receptors transduce intracellular signals through various mechanisms. EP1 is linked to the mobilization of Ca^2+^. EP2 and EP4 stimulate adenylate cyclase, while EP3 inhibits adenylate cyclase [133]. PGE2 allows the nociceptor neurons to become sensitive to pain and other stimuli. The action of PGE2 occurs mainly on proximal ion channels and acts as a sensitizer for nociceptor activity, instead of as a direct activator of nociceptive neurons. This process is the key to understanding the analgesic effect and the analgesic power of NSAIDs [134]. PGE2 may induce persistent hyperalgesia via the PKA and PKC-mediated activation of NF-κB in DRG neurons if it is present over a long period of time [135]. Prostaglandins are not the only class of lipids involved in inflammation. There are several classes of lipid molecules with both pro- and anti-inflammatory properties that are, respectively, involved in the activation or silencing of nociceptor activity. Some molecules, such as lysophosphatidic acid and sphingosine-1-phosphate, have a direct effect on nociceptor neurons during inflammation and increase TRPV1 activity [136,137]. Another example is leukotriene B4 (LTB4), which induces hyperalgesia in humans if injected [138] through its activity in the modulation of C-fibers and Ad-fibers [139]. BLT1, the receptor for LTB4, is expressed by a subset of TRPV1^+^ DRG neurons. Its role consists of mediating calcium flux after ligand activation [140]. Several studies demonstrate that some anti-inflammatory and pro-resolving lipids may silence pain through their activity. Pain can be blocked by the anti-inflammatory prostaglandin J2 (PGJ2), which activates PPARγ, and, as a consequence, indirectly activates K + ATP channels in nociceptors [141]. Lipoxins, resolvins, protectins, and maresins are classified as pro-resolving lipids and have been demonstrated to have analgesic effects [142,143]. One example is the lipoxin receptor ALXR/FPR2, which is expressed by spinal cord astrocytes. In this case, lipoxin A4 inhibits ERK and JNK activation in astrocytes and is transduced as a decrease in inflammatory pain. Receptor (ChemR23) of resolvin E1 (RvE1) is expressed by TRPV1-positive neurons [144]. The activity of RvE1 is due to the inhibition of ERK, which is induced by TNF/α and capsaicin in these DRG and spinal cord neurons [145].

**Figure 2:**
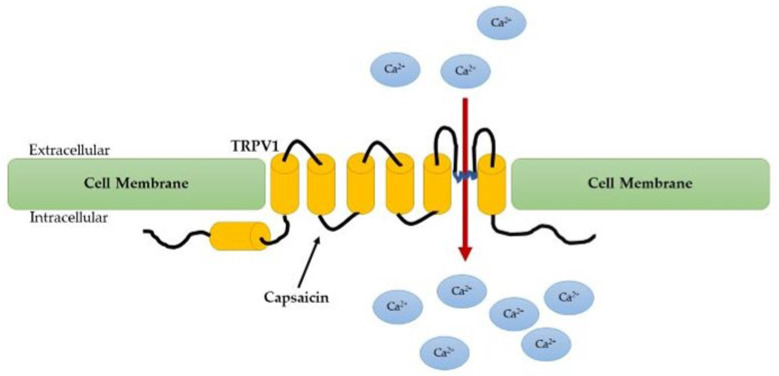
Capsaicin activates the TRPV1 on sensory neurons to alter their membrane potential and induce pain.

Capsaicin is a natural product present in hot chili peppers and is the active compound found in many spicy foods. The exposure of nociceptor terminals to capsaicin leads to an excitation of neurons with a perception of pain and local release of inflammatory mediators. If the exposure is prolonged, nociceptor terminals become insensitive to capsaicin and other stimuli [146]. Capsaicin-sensitive peptidergic sensory nerves mediate pain (classical afferent function) but, moreover, play an important role in inflammation via sensory neuropeptide release (efferent function). TRPV1 is located on these nerves and is a non-selective cation channel that is activated and sensitized by several irritants, including capsaicin, resiniferatoxin (RTX), and endogenous molecules such as protons, bradykinin, prostanoids, TNF/α, nerve growth factor, gasotransmitters, and lipid peroxidase products [147] (Figure 2).

With the activation of capsaicin-sensitive nerve terminals, several sensory neuropeptides, such as substance P, neurokinin A, and calcitonin gene-related peptide that lead to vasodilation and inflammatory cell recruitment (neurogenic inflammation) [148,149,150,151,152] and have a potent anti-inflammatory and antinociceptive action, are released. The long exposure to capsaicin and subsequent desensitization of the receptor may be explained by the death of the nociceptor or the destruction of its peripheral terminals [148,149,150,151]. Capsaicin can provoke the perception of pain through its action on nociceptors, which may mimic the action of a physiological stimulus or an endogenous ligand produced during tissue injury [153].

The effect of these processes is a reduction in the release of excitatory glutamate, which leads to a reduction in pain sensitivity [143]. The role of protectin D1 is to inhibit the neuronal plasticity caused by TRVP1 activation and TNF/α [145]. Furthermore, TRPV1 currents in DRG are inhibited by maresin 1, which reduces inflammatory pain [143]. Neither protectin D1 nor maresin 1 influence nociceptive mechanisms downstream of TRPA1 [115,145]. These results, when considered together, may open a new viewpoint towards the role of pro-inflammatory lipids, such as PGE2 and LTB4, in the activation of pain. According to the results of these studies, lipid mediators play an important role in silencing nociceptor neuron sensitization and activation. The future possibility of pain being treated with the induction or administration of PGJ2 and pro-resolving lipids cannot be excluded.

## 8. Nerve Injury and Neuropathic Pain

Trauma, metabolic imbalance, viral infection, and chemotherapeutic agents all cause injury to the nervous system. The pain associated with these types of injury is termed neuropathic pain. All forms of neuropathic pain share a common mechanism, even if the injury causing it is different in nature or modality [154]. For instance, after nerve injury caused by trauma, there is a loss of trophic factors that leads to a change in neurotransmission because of the modified expression of ion channels which change in density and distribution. This is transduced as an increased excitation in injured afferents [154]. This injured nerve does not work properly, however, and there is, therefore, a loss of competition with other afferents for trophic factors released from peripheral targets. This leads to a greater availability of trophic factors for uninjured neighbors instead of for the injured afferents [155]. A phenotypic change may be induced by the presence of greater quantities of available trophic factors, which is transduced as an increase in excitability [155]. There is still an open debate about the actual contribution of injured and uninjured afferents in the pain associated with traumatic nerve injury. As previously mentioned, neuropathic pain may have different causes, as well as several common characteristics, such as ongoing pain [156]. Unlike inflammatory pain, in this type of pain, the elimination, if possible, of the stimuli that affect the inflamed tissue does not alleviate the pain. Further investigation of neuropathic pain is required to better understand the basis of the mechanisms and pathways of the ongoing pain in the absence of stimuli. For this type of pain, the area of study concentrates primarily on the afferent aspect as it has been shown that the administration of some pharmaceuticals, such as local anesthetics, are able to alleviate ongoing neuropathic pain [157]. The ongoing afferent activity may act in different ways in order to induce changes in transduction. The mechanisms can vary and may include the expression of transducers in neurons that normally do not express this type of transducer, the increase in expression of excitatory receptors [158], and/or the decrease of inhibitory transducers [159]. Another mechanism may be the expression of thermal or mechanical transducers near the extremity of the cut, damaged axon [159], or inside the ganglia [160]. It is plausible to hypothesize that the various processes occur and collaborate simultaneously to contribute to the ongoing activity in the afferents affected during nerve injury. The origins of the activity may include, as previously mentioned, the ectopic expression of transducers [161]. One example is the anomalous activation of nociceptors by norepinephrine which results from the sympathetic post-ganglionic terminals that are expressed on ganglia [162] and the alteration in expression and density of ion channels that leads to instability and spontaneous activity on the membrane [163]. These mechanisms of activity are not only a consequence of the damage but are likely to be a result of the various changes that occur over time. For these reasons, neuropathic pain is difficult to manage.

## 9. Neuronal Regulation of Vasculature

Neuronal regulation of vasculature and inflammation is demonstrated with experiments that show redness, heat, and swelling independent of the sensory nerve supply [144]. Neurogenic inflammation is a process based on direct electrical nerve stimulation which produces vasodilation and permeability [164]. This process is a possible mechanism for the mediation of axon–axon reflexes that transduce the signal in neighboring axons through calcium influx and antidromic signaling and causes the release of mediators stored in vesicles located at the axon terminals in the periphery. Several nociceptor mediators are recognized, including the neuropeptides calcitonin gene-related peptide and substance P, which are potent mediators of vasodilation and tissue edema [150,165,166].

Substance P plays a key role in several brain disorders and the pain response following different types of inflammation [167]. Several experiments have been conducted on the modulation and transmission of pain caused by substance P [168,169,170,171]. Substance P is a peptide that is made up of 11 amino acids and along with others, such as neurokinin A, neurokinin B, neuropeptide-γ (NPγ), and hemokinin 1, makes up a part of the tachykinin family. Substance P can be found in different fiber types and districts, including in unmyelinated C fibers, primary afferent fibers in both the peripheral and central nervous system, primary sensory afferent fibers, the dorsal horn of the spinal cord, and dorsal root ganglia, and is released from non-neuronal cells, such as inflammatory and endothelial cells [172,173]. Substance P is also located in neurons that are sensitive to capsaicin [152], where it is released following various chemical, thermal, and mechanical stimuli and can be activated by ligand binding [174]. Substance P and other tachykinin neuropeptides are able to bind NK1, NK2, and NK3 G-protein-coupled receptors. NK1 is generally expressed at elevated concentrations in both the brain and peripheral tissues. Substance P has the highest affinity for the NK1 receptor [175], which is strongly expressed in the brain rendering substance P of particular interest in the study of pain in the central nervous system. Substance P and calcitonin gene-related peptide have a direct action on smooth muscle cells and vascular endothelial cells in the mediation of neurogenic inflammation.

The release of substance P and calcitonin gene-related peptide is also associated with migraines. It is hypothesized [176] that a massive release of serotonin from the median raphe is correlated with the activation of serotonergic receptors located on the walls of large cerebral vessels. This may lead to an increase in the transmural pressure of these vessels and increases vasodilatation. The increase in transmural pressure leads to the activation of the trigeminal nerve with consequent antidromic stimulation of the sensory nerves that is translated into the consequent release of pro-inflammatory peptides (substance P and calcitonin gene-related peptide) at the level of hard vessels in the meninges [177,178]. Substance P also acts on lymphatic vessel contractility, thereby increasing pump efficiency stimulating its receptors TACR1 and TACR3, which are expressed on lymphatic smooth muscle cells [179,180,181]. RAMP1 is the receptor for calcitonin gene-related peptide. It is involved in angiogenesis and lymphangiogenesis during skin injury healing and is necessary for the regulation of VEGF production [105]. Although there is evidence of interaction between the nociceptive system and blood or lymphatic vessels, it remains to be seen whether or not this exchange of interactions regulates antigen drainage and adaptive immunity.

## 10. Conclusions

It is clear that nociceptive neurons and the immune system play a central role in pain and inflammation. The function of the immune and nociceptive systems is based on recognizing damaging and/or harmful stimuli. Their response plays an important role in preventing tissue damage and restoring homeostasis. The dysregulation of these interactions may underlie the pathogenesis of several inflammatory diseases. The interactions between the immune system and nociceptive neurons occur within both peripheral sites of injury as well as in the central nervous system. The modulation of nociceptive neuron activity and its mediators may provide new approaches to the treatment of pain and chronic inflammatory disease. The role of the sensory nervous system is key to the modulation of the host’s protective response. Understanding its interactions is crucial to revealing new strategies for the treatment of pain. To date, current therapies often lack the desired level of efficacy or tolerability necessary to provide optimal pain management. The goal of future research will be to obtain a greater understanding of ion channel modulation so that it can be exploited as a fundamental resource in the quest for the development of the next generation of pain modulation drugs.

## Figures and Tables

**Table 1:** Mediator and receptors associated with immune cells.

Immune Cell	Mediator	Receptor
Mast cell	IL-5	IL-5R
	5-HT	5-HT2
	IL-6	Gp130
	IL-1β	IL-1R1
	TNF/α	TNFR1
	Histamine	H1/2
	NGF	TrkA
T cell	IL-17A	IL-17AR
	IFN-γ	IFN-γR

## Data Availability

Not applicable.
